# Rapidly Progressive Dedifferentiated Liposarcoma of the Spermatic Cord: A Case Report

**DOI:** 10.3390/ijms27209172

**Published:** 2026-10-16

**Authors:** Franky Rocha, Jair Figueroa-Emiliani, Elizabeth Vargas-Castellanos

**Affiliations:** Hospital Universitario Mayor-Méderi, Universidad del Rosario, Bogota 111411, Colombia

**Keywords:** dedifferentiated liposarcoma, spermatic cord, paratesticular tumor

## Abstract

Dedifferentiated liposarcoma (DDLPS) of the spermatic cord is an exceptionally rare and aggressive malignancy for which diagnostic and therapeutic guidelines remain limited. We report the case of a 73-year-old man who presented with a rapidly enlarging left inguinal/paratesticular mass. Initial ultrasonography showed a 4.3 × 3.0 × 3.7 cm hypoechoic lesion interpreted as suggestive of leiomyoma. Pelvic magnetic resonance imaging subsequently demonstrated a 10 × 5 × 5 cm neoplastic mass arising from the spermatic cord. The patient underwent radical left orchiectomy. Histopathological examination revealed a high-grade spindle-cell neoplasm with marked pleomorphism and up to 30 mitoses per 10 high-power fields. Immunohistochemistry showed positivity for p16, MDM2, and CDK4, with a Ki-67 proliferative index of 95%, supporting the diagnosis of high-grade DDLPS. Although surgical margins were negative, the disease progressed rapidly. Nineteen days after surgery, the patient developed bilateral pleural effusion, pleural infiltration, pulmonary metastases, and a probable pathological T11 fracture. Because of extensive metastatic disease and clinical deterioration, systemic antineoplastic therapy was not initiated in this advanced setting. The patient died 27 days after hospitalization due to progressive metastatic disease. This case highlights the diagnostic difficulty, aggressive biological behavior, and need for early multidisciplinary management of spermatic cord DDLPS.

## 1. Introduction

Liposarcomas (LPSs) are malignant neoplasms of mesenchymal origin characterized by adipocytic differentiation and constitute one of the main subtypes of soft tissue sarcomas. The LPS comprises four main subtypes: well-differentiated liposarcoma (WDLPS) or atypical lipomatous tumor, dedifferentiated LPS (DDLPS), myxoid LPS, and pleomorphic LPS [1]. DDLPS is a rare and particularly aggressive neoplasm characterized by areas of WDLPS associated with a high-grade, non-lipogenic differentiation component [2]. It is predominantly located in the retroperitoneum and extremities, although it can develop in less common anatomical sites. Its presentation in unusual locations poses a diagnostic challenge, making the integration of clinical, radiological, and histopathological findings essential for establishing an accurate diagnosis [3].

DDLPS is characterized by the presence of a well-differentiated lipomatous component associated with a variable proportion of non-lipogenic, morphologically distinct dedifferentiated areas. The dedifferentiated component may display a broad histological spectrum, ranging from relatively uniform spindle-cell proliferation to highly pleomorphic and anaplastic sarcoma. This morphological heterogeneity may complicate diagnosis, particularly in tumors arising at unusual anatomical sites. In contrast to WDLPS, which generally demonstrates limited metastatic potential, DDLPS is associated with a substantially higher risk of aggressive local behavior and distant dissemination [2,3].

At the molecular level, both WDLPS and DDLPS are characterized by amplification of the chromosome 12q13–15 region, which includes the MDM2 and CDK4 genes. MDM2 amplification plays an important role in the disruption of the p53 tumor suppressor pathway, whereas CDK4 amplification contributes to dysregulation of cell-cycle progression. Detection of these alterations, particularly MDM2 and CDK4 amplification or overexpression, can therefore provide valuable diagnostic support in challenging cases and may have therapeutic implications in advanced disease [2,3].

Malignant paratesticular tumors are uncommon, accounting for only 7–10% of scrotal masses; therefore, their natural history and optimal treatment remain insufficiently studied [3]. LPS accounts for approximately 21% to 51% of all paratesticular sarcomas, with the spermatic cord being the most affected structure. The mean age at presentation is approximately 60–65 years [2]. These tumors exhibit a wide histological spectrum and may clinically resemble benign conditions such as lipomas, epididymal cysts, or inguinal hernias, sometimes resulting in a delayed or inadvertent diagnosis. Their biological behavior is aggressive, with a significant risk of recurrence and metastasis [4,5].

Given the limited evidence available to guide the management of spermatic cord DDLPS, we report the case of a 73-year-old man with high-grade DDLPS originating in the left spermatic cord. The tumor showed rapid clinical growth, marked histological atypia, high mitotic activity, and a Ki-67 proliferative index of 95%. Despite complete surgical resection with negative margins, the patient developed extensive pleuropulmonary metastatic disease shortly after surgery and died from rapidly progressive disease. This case illustrates the diagnostic challenges associated with DDLPS at an uncommon paratesticular site and highlights the importance of early staging, multidisciplinary assessment, and close postoperative surveillance.

## 2. Case Report

A 73-year-old man presented to the emergency department of Hospital Universitario Mayor-Méderi (Bogotá, Colombia) with a mass in the left inguinal canal, near the ipsilateral testicle, that had been present for approximately two months. The patient reported progressive growth of the lesion, associated with occasional local pain. He brought a testicular ultrasound performed outside the hospital, which identified a hypoechoic lesion with well-defined borders, measuring approximately 4.3 × 3.0 × 3.7 cm, initially interpreted as suggestive of a leiomyoma. Physical examination revealed a palpable mass along the left spermatic cord, approximately 7 × 5 cm, with irregular borders, firm consistency, and no signs of fluctuation. The left testicle was unremarkable on physical examination. The patient had no relevant medical history. The patient’s COVID-19 vaccination status was not documented in the available medical records. Therefore, his vaccination history could not be retrospectively established.

**Figure 1:**
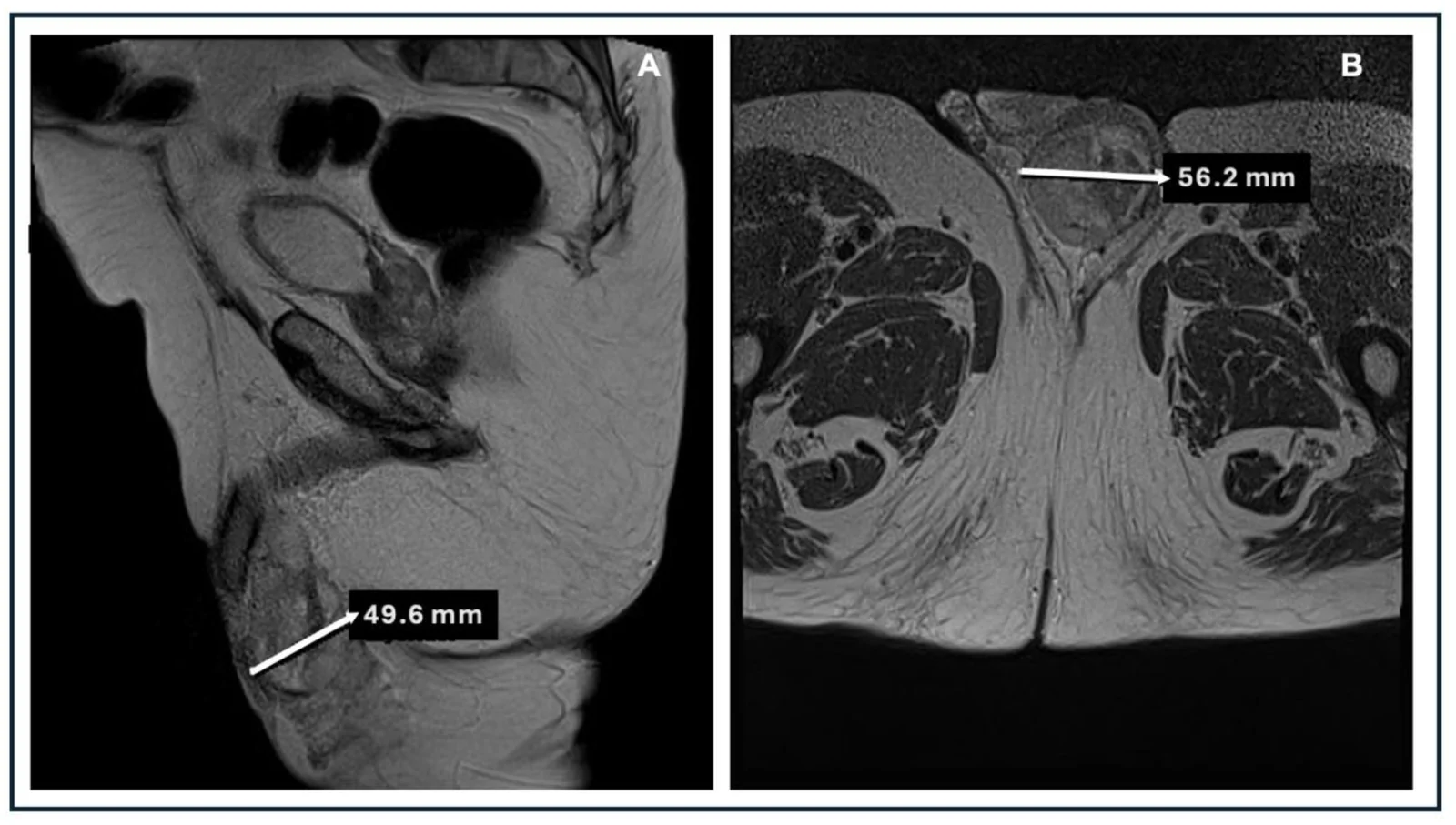
Contrast-enhanced magnetic resonance imaging of the pelvis. (**A**) Sagittal image showing a left paratesticular mass extending into the spermatic cord, with a representative measurement of approximately 5.0 cm. (**B**) Axial image showing the same mass, with a representative measurement of approximately 5.6 cm, with diffusion restriction and heterogeneous enhancement following intravenous contrast administration.

Given the rapid increase in size, a pelvic magnetic resonance imaging was requested, which revealed a mass approximately 10 × 5 × 5 cm, located in the left paratesticular region and likely originating from the spermatic cord, with characteristics suggestive of a neoplastic lesion (Figure 1). As part of the diagnostic approach, tumor markers were ordered. The beta subunit of human chorionic gonadotropin (β-hCG), measured qualitatively, was positive. Serum levels of lactate dehydrogenase (LDH) were 242.30 U/L (reference range: 120–246 U/L) and alpha-fetoprotein (AFP) were 0.00 ng/mL (reference range: up to 4 ng/mL), both within normal limits.

The patient underwent a left radical orchiectomy as definitive surgical treatment. During the procedure, a tumor mass adjacent to the testicle, originating from the spermatic cord, was identified.

Macroscopic pathological examination revealed a multilobulated, whitish mass with areas of hemorrhage, originating from the spermatic cord. The tumor measured 8.5 × 5 × 5 cm and was located approximately 1 cm from the spermatic cord resection margin. The testicular parenchyma was free of tumor infiltration. Histopathological examination showed a neoplasm composed of interlacing fascicles of spindle-shaped cells, associated with areas of large, pleomorphic cells, some displaying marked nuclear atypia. Up to 30 mitotic figures were identified per 10 high-power fields, with no evidence of tumor necrosis. The spermatic cord resection margin was negative for malignancy.

**Figure 2:**
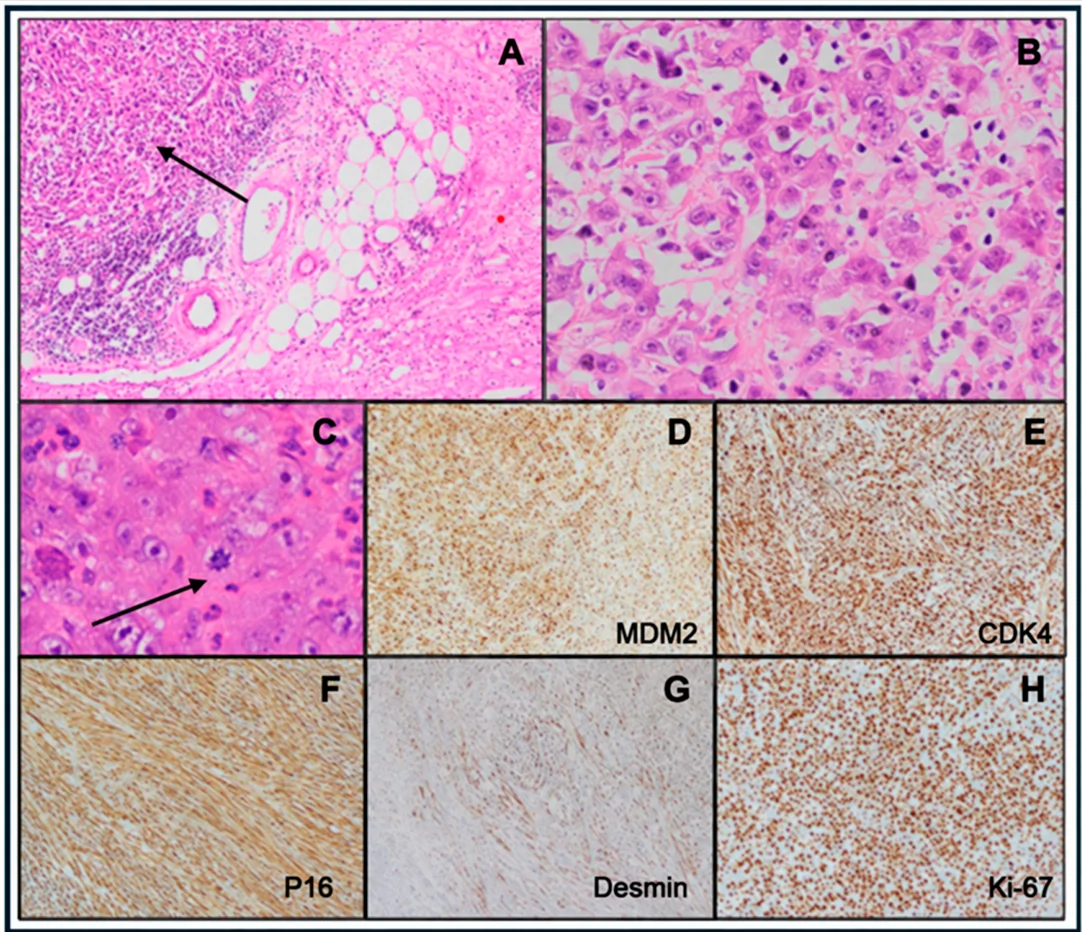
Histopathological and immunohistochemical findings of dedifferentiated liposarcoma of the spermatic cord. (**A**) H&E, ×4. Low-power view of a mesenchymal neoplasm composed of spindle cells arranged in interlacing fascicles, with entrapped adipose tissue and areas containing large, atypical cells (arrow), without evidence of tumor necrosis. (**B**) H&E, ×10. Higher-magnification view showing markedly atypical spindle cells with prominent pleomorphism, enlarged irregular nuclei, and conspicuous nucleoli. (**C**) H&E, ×10. Atypical mitotic figure within the dedifferentiated component (arrow). (**D**) IHC, ×4. Positive nuclear staining for MDM2. (**E**) IHC, ×4. Positive nuclear staining for CDK4. (**F**) IHC, ×4. Positive staining for p16. (**G**) IHC, ×4. Patchy positive staining for desmin. (**H**) IHC, ×4. Ki-67 proliferation index of approximately 95% in the dedifferentiated component.

Immunohistochemical staining showed positivity for p16 (Figure 2F), murine double minute 2 (MDM2) (Figure 2D), and CDK4 in the neoplastic cells (Figure 2E), with focal or patchy expression of cytokeratin, smooth muscle actin, S100, and desmin (Figure 2G). CD34 and STAT6 markers were negative. The cell proliferation index, assessed by Ki-67, was 95% (Figure 2H). Overall, the morphological and immunohistochemical findings were consistent with a diagnosis of high-grade DDLPS of the spermatic cord. The postoperative period was uneventful, and the patient was discharged with outpatient follow-up.

Nineteen days after surgery, the patient was readmitted to the hospital with dyspnea and pleuritic chest pain. As part of the staging workup, bilateral pleural effusion, predominantly on the right, was documented, associated with bilateral infiltration of the visceral and parietal pleura, as well as bilateral pulmonary metastasis. Additionally, an incomplete burst fracture of the T11 vertebral body was identified, considered likely to be of pathological origin in the context of advanced neoplastic disease.

During hospitalization, thoracentesis was performed, yielding pleural fluid with hemorrhagic and exudative characteristics, consistent with pleural effusion associated with the neoplastic disease. The patient was evaluated by the Oncology service, which, considering the histological diagnosis, the metastatic extent of the disease, and the significant deterioration of the patient’s general condition, determined that there was no expected clinical benefit from initiating systemic antineoplastic treatment or palliative oncological measures aimed at disease control.

After 27 days of hospitalization, the patient died because of complications related to the progression and metastatic involvement of the DDLPS.

## 3. Discussion

Paratesticular malignancies are uncommon, and LPS is the most common sarcoma arising in the spermatic cord [2,4]. In a retrospective analysis of 265 reported cases of paratesticular LPS, the median patient age was 62 years, and the median tumor size was 9.5 cm. Among these patients, 178 underwent high inguinal orchiectomy, whereas 40 underwent simple tumorectomy [5]. The patient’s age (73 years) and the size of the resected tumor (8.5 cm) fall within this range. However, the exceptionally rapid tumor growth, high histological grade, and unfavorable clinical course distinguish this case from the less aggressive presentations described previously.

Paratesticular liposarcomas may arise from the adipose tissue surrounding the spermatic cord. Although malignant transformation of a pre-existing lipoma has been proposed, the progression from lipoma to liposarcoma remains poorly established, and some lesions initially diagnosed as lipomas may instead represent an atypical lipomatous tumor or a WDLPS that was misclassified because of sampling limitations or diagnostic challenges [6,7]. Amplification of the *MDM2* gene, typically assessed by fluorescence in situ hybridization (FISH), is a key molecular hallmark of Atypical Lipomatous Tumor (ALT)/WDLPS and, together with *CDK4* amplification, provides important diagnostic support. In a series of 439 mature lipomatous lesions evaluated by *MDM2* FISH, 17 of 75 tumors (22.6%) ultimately classified as ALT/WDLPS lacked cytological atypia on the initial histological assessment and had therefore favored a diagnosis of lipoma [8]. This finding is particularly relevant in the spermatic cord, where lipomatous lesions may be clinically interpreted as cord lipomas or inguinal hernias [6].

In our patient, the mass had been noticed only two months before presentation, and the resected tumor was predominantly composed of a non-lipogenic, high-grade component with entrapped adipose tissue (Figure 2A,B). Although molecular confirmation of *MDM2* and *CDK4* amplification would have provided additional diagnostic support, these analyses could not be performed in our setting. In Colombia, access to molecular testing for uncommon tumors remains limited, and such assays may require specific authorization, are not consistently covered by the healthcare system, and may involve prolonged processing times. Consequently, the diagnosis in this case was based on the integrated clinical, morphological, and immunohistochemical findings. When the well-differentiated component is scant or not represented in the sampled tissue, DDLPS may be difficult to recognize as an adipocytic neoplasm on morphology alone, making ancillary molecular studies particularly valuable when available.

These tumors typically present as firm, unilateral, slowly enlarging, painless masses and may be clinically mistaken for more common conditions, including spermatic cord cysts, hydrocele, epididymitis, or inguinal hernia, potentially delaying definitive diagnosis and treatment [9]. Our 73-year-old patient, however, presented with a markedly different clinical course, characterized by rapid tumor enlargement. The lesion approximately doubled in size over three months and measured 8.5 × 5 × 5 cm at pathological examination. Similar patterns of rapid growth have been reported in patients with DDLPS of the spermatic cord. Sambel et al. described a 66-year-old man with a six-month history of a scrotal mass that underwent marked enlargement during the preceding two months, reaching 11.5 × 5.3 × 6.3 cm on magnetic resonance imaging (MRI) [10]. Likewise, Ando et al. reported a 62-year-old man with a painful spermatic cord DDLPS that developed over approximately three months and measured approximately 10 cm [11]. Collectively, these cases suggest that, although spermatic cord liposarcomas may initially manifest as slowly enlarging masses, DDLPS can exhibit rapid tumor progression and a clinically aggressive course.

The imaging findings in our patient further illustrate the diagnostic challenges associated with paratesticular and spermatic cord sarcomas. Ultrasonography is useful for distinguishing extratesticular from intratesticular lesions, whereas computed tomography (CT) and MRI provide more detailed assessment of tumor size, margins, tissue composition, and local extension along the spermatic cord, including involvement beyond the deep inguinal ring into the pelvis or retroperitoneum [12]. Although the radiological features of paratesticular sarcomas are generally similar to those of sarcomas arising at other anatomical sites, their rarity in this location may increase the likelihood of misinterpretation as benign or other more common lesions. In the present case, the initial ultrasonographic examination was interpreted as a leiomyoma, highlighting the difficulty of distinguishing a malignant spermatic cord neoplasm from other solid lesions, including smooth-muscle tumors such as leiomyosarcoma [13]. This diagnostic challenge may be particularly relevant in DDLPS, which can contain a substantial non-lipomatous component and therefore lack the characteristic macroscopic fat that may suggest an adipocytic tumor. In our patient, MRI subsequently demonstrated heterogeneous enhancement and diffusion restriction, findings that raised greater concern for a malignant neoplasm. The absence of a primary intratesticular lesion and normal AFP and LDH levels further argued against a germ cell tumor. Although β-hCG was qualitatively positive, it was neither quantified nor repeated; therefore, its clinical significance remains uncertain, and this isolated finding was considered nonspecific. Overall, this case underscores the limitations of initial imaging in this anatomically uncommon location and the importance of considering malignancy when evaluating solid paratesticular or spermatic cord masses, particularly when imaging features are atypical for a benign lesion.

Macroscopically, DDLPS may present as lobulated or nodular masses ranging from 3 to 30 cm, with a variable gray-white to yellow-brown appearance and, in some cases, areas of hemorrhage or necrosis [14]. In our patient, the resected specimen measured 8.5 cm and was predominantly composed of a non-lipogenic, high-grade component with entrapped adipose tissue. Histologically, DDLPS is characterized by a dedifferentiated non-lipogenic component arising in association with a well-differentiated liposarcoma component, with dedifferentiated areas often resembling high-grade pleomorphic or spindle-cell sarcoma and showing marked cytological atypia and increased mitotic activity [14]. Consistent with these features, our specimen showed a high-grade spindle-cell proliferation with marked pleomorphism and increased mitotic activity.

Immunohistochemical positivity for MDM2 and CDK4 supported the diagnosis of high-grade DDLPS, while the very high Ki-67 labeling index (95%; Figure 2H) indicated a markedly proliferative tumor phenotype. Although the Ki-67 labeling index may vary considerably among DDLPS, markedly elevated proliferative indices have been reported in aggressive tumors, including approximately 80% in a DDLPS of the esophagus, 70–80% in a DDLPS with heterologous myogenic differentiation and testicular metastasis, and approximately 80% in a recurrent scrotal DDLPS with epithelioid features [15,16,17]. The Ki-67 index of 95% observed in our patient therefore represents a markedly elevated proliferative fraction and is consistent with the high-grade morphology and aggressive clinical behavior observed in this case.

Histologically, DDLPS is characterized by the coexistence of a well-differentiated lipogenic component and a non-lipogenic dedifferentiated component, which may range from relatively low-grade spindle-cell proliferation to high-grade pleomorphic sarcoma [2,3]. In our patient, the dedifferentiated component was at the high-grade end of this spectrum, showing fascicles of atypical spindle cells, marked cellular pleomorphism, and up to 30 mitoses per 10 high-power fields. According to the Fédération Nationale des Centres de Lutte Contre le Cancer (FNCLCC) grading system, the tumor was classified as grade 3, based on a differentiation score of 3, a mitotic score of 3, and a necrosis score of 0 [18]. In the spermatic cord, the differential diagnosis of a predominantly non-lipogenic spindle- and pleomorphic-cell neoplasm includes leiomyosarcoma, undifferentiated pleomorphic sarcoma, solitary fibrous tumor, and sarcomatoid carcinoma. In this case, the immunohistochemical profile helped narrow these possibilities: the absence of STAT6 and CD34 expression argued against solitary fibrous tumor, while only focal desmin and smooth muscle actin expression did not support leiomyosarcoma. Similarly, focal cytokeratin expression in the absence of a recognizable epithelial component was insufficient to support sarcomatoid carcinoma. Importantly, the co-expression of MDM2, CDK4, and p16 provided additional support for an MDM2-associated adipocytic neoplasm [2,3,19]. However, immunohistochemical expression of MDM2 and CDK4 does not establish gene amplification, and molecular testing would have provided additional diagnostic confirmation [20]. Such testing was not available in our case, representing an important limitation of the report. In our setting, access to molecular testing for uncommon tumors may be constrained by authorization requirements, limited availability, and prolonged processing times, which can preclude timely testing in routine clinical practice. Nevertheless, molecular characterization remains valuable not only for diagnostic confirmation but also because MDM2-amplified DDLPS is currently being investigated as a target for MDM2-directed therapies, further supporting the use of molecular testing whenever it is accessible [21,22].

The most distinctive feature of this case was the rapid systemic progression despite margin-negative surgical resection. In a large clinicopathological series, DDLPS recurred locally in approximately 40% of patients and metastasized in about 17%, with disease-related mortality of approximately 28% [23]. In paratesticular liposarcoma, local recurrence rather than distant dissemination is generally the predominant pattern of failure. In a series of 25 patients, more than half of whom had DDLPS, only one developed distant metastasis, whereas local recurrences frequently occurred along the gonadal vessels and were strongly associated with positive surgical margins [24]. Similarly, the pooled analysis by Kamitani et al. identified dedifferentiated, pleomorphic, and round-cell liposarcomas as the subtypes associated with the highest risk of recurrence [5]. When DDLPS metastasizes, the lungs are among the most frequent sites, with a reported median interval of approximately 25 months from primary diagnosis to metastasis and a median survival of about one year after metastatic disease is identified [25]. Unusual metastatic sites have also been described in paratesticular liposarcoma, including the penile crus [26].

Our patient showed a substantially more aggressive course. He underwent radical left orchiectomy with proximal mobilization and division of the spermatic cord at the level of the deep inguinal ring, and an enlarged left inguinal lymph node was also excised. Macroscopic pathological examination showed that the tumor measured 8.5 × 5 × 5 cm and was located approximately 1 cm from the proximal spermatic cord resection margin. Histopathological examination confirmed a negative spermatic cord resection margin and no lymph node involvement. Despite the negative margin, metastatic disease was documented only 19 days after surgery, involving both lungs and the visceral and parietal pleura, with a hemorrhagic pleural effusion and possible vertebral involvement. This exceptionally short interval makes it unlikely that clinically significant metastatic disease developed entirely after surgery. Instead, it suggests that occult systemic dissemination was already present at the time of the initial operation, although the exact timing of metastatic spread cannot be established.

Importantly, adjuvant radiotherapy was not administered before the patient’s rapid clinical deterioration. The available medical record does not document a specific postoperative decision against radiotherapy or provide a formal assessment of its potential benefit. Notably, in the largest retrospective analysis of paratesticular liposarcoma, adjuvant radiotherapy after resection was not associated with a statistically significant improvement in recurrence-free survival, including among patients with positive margins [5]. Rather, the patient was readmitted 19 days after surgery with extensive pleuropulmonary metastatic disease and a markedly compromised clinical status. At that time, oncology considered that, given the histological subtype, advanced tumor burden, and overall condition, he would not benefit from antineoplastic systemic treatment and recommended supportive and palliative management. Thus, the absence of adjuvant radiotherapy should not be interpreted as the cause of the subsequent rapid progression; instead, the early recognition of disseminated disease effectively precluded postoperative adjuvant treatment. Given the high histological grade of the primary tumor, these findings also underscore the importance of comprehensive preoperative staging and multidisciplinary evaluation, including consideration of adjuvant treatment when clinically appropriate.

For advanced or metastatic DDLPS, systemic therapy is primarily palliative, and treatment remains challenging. Doxorubicin-based chemotherapy remains the standard first-line option, with ifosfamide- or gemcitabine-based regimens considered in selected patients, although objective responses in DDLPS are relatively uncommon [27]. Several agents have subsequently demonstrated activity in advanced liposarcoma. Trabectedin improved progression-free survival compared with dacarbazine in a randomized phase III trial of patients with advanced liposarcoma or leiomyosarcoma previously treated with anthracyclines, with the benefit observed across prespecified subgroups [28]. Similarly, eribulin significantly improved overall survival compared with dacarbazine in a phase III trial of previously treated advanced liposarcoma or leiomyosarcoma, although its use may be associated with substantial treatment-related toxicity [29]. Pazopanib has also demonstrated clinical activity in advanced liposarcoma, although the magnitude of benefit appears modest and its role is less well established in DDLPS specifically [30].

More recently, therapeutic strategies have increasingly incorporated the molecular characteristics of DDLPS. These tumors are frequently characterized by *MDM2* and *CDK4* amplification, which has prompted investigation of subtype-directed therapies targeting these pathways. Selinexor, an oral selective inhibitor of exportin 1 (XPO1), represents a related molecularly informed approach and has been evaluated specifically in DDLPS. In the phase II/III SEAL trial, patients with previously treated advanced DDLPS experienced a statistically significant improvement in progression-free survival with selinexor compared with placebo (median, 2.8 vs. 2.1 months; HR, 0.70), although the absolute benefit was modest. No overall survival advantage was observed, although interpretation of this endpoint was potentially influenced by the permitted crossover from placebo to selinexor [31]. Similarly, the recently reported phase II/III Brightline-1 trial evaluated the oral MDM2–p53 antagonist brigimadlin versus doxorubicin as first-line treatment in patients with locally advanced or metastatic, MDM2-amplified DDLPS. Although brigimadlin demonstrated antitumor activity, with a higher objective response rate and evidence of TP53 pathway activation, the primary endpoint of progression-free survival was not met. Median progression-free survival was 8.4 months with brigimadlin versus 7.2 months with doxorubicin (HR, 0.79; 95% CI, 0.60–1.06; *p* = 0.096). These findings underscore both the biological rationale for MDM2–p53 targeting in DDLPS and the challenges of translating molecularly defined vulnerabilities into clinically superior first-line therapies, indicating that the role of MDM2 inhibition in DDLPS remains under investigation [21]. Overall, these findings illustrate both the potential and the limitations of systemic and molecularly informed therapies, most of which provide disease control rather than durable tumor regression.

Importantly, none of these trials was designed specifically for spermatic cord DDLPS, and most evidence for systemic therapy derives from patients with advanced liposarcoma treated in specialized oncology settings. Their applicability therefore remains uncertain in this rare anatomical presentation and, particularly, in patients with poor performance status and rapidly progressive disease [32]. In our patient, the hemorrhagic pleural effusion, bilateral pulmonary metastases, probable pathological vertebral involvement, and rapid functional decline left no realistic therapeutic window. Although doxorubicin would have represented the systemic option supported by the most established first-line evidence, the oncology team considered that the advanced tumor burden and compromised clinical condition made antineoplastic treatment unlikely to provide meaningful benefit and therefore prioritized supportive and palliative care [27,33]. This case illustrates a clinical scenario poorly represented in therapeutic trials: high-grade DDLPS with exceptionally rapid systemic progression before systemic treatment could be meaningfully started.

Taken together, comparison with the published literature suggests that this case represents an uncommon and particularly aggressive presentation of spermatic cord DDLPS [5,23]. In contrast to the more indolent, predominantly locally recurrent course generally described for paratesticular liposarcoma, our patient presented with a short history of rapid tumor growth, high-grade dedifferentiated morphology, a markedly elevated Ki-67 index of 95%, and pleuropulmonary metastatic disease with suspected vertebral involvement documented only 19 days after margin-negative resection. This exceptionally rapid clinical course suggests that occult systemic dissemination was likely already present at the time of surgery. However, the timing of metastatic spread cannot be established with certainty. Practical lessons from this case include maintaining a high index of suspicion for malignancy in patients with a rapidly enlarging or painful spermatic cord mass, even when initial ultrasonography suggests a benign lesion; obtaining comprehensive preoperative staging, including thoracic imaging, before definitive surgery; considering molecular confirmation of MDM2 amplification by FISH, particularly when the well-differentiated component is scant or not readily identifiable; and referring patients early to a multidisciplinary sarcoma team. Given the rarity of spermatic cord DDLPS and the paucity of cases with early distant dissemination, pooled analyses of patients with paratesticular DDLPS and metastatic disease may help identify clinical, histopathological, and molecular features associated with this particularly aggressive behavior.

## 4. Conclusions

DDLPS of the spermatic cord is a rare and aggressive malignancy that may present with rapid tumor growth and a substantial risk of early metastatic progression. Because paratesticular sarcomas can mimic benign lesions on initial imaging, a high index of suspicion is required when evaluating rapidly enlarging or atypical paratesticular masses. Definitive diagnosis relies on careful histopathological assessment complemented by immunohistochemical studies, including MDM2 and CDK4 expression, while molecular testing may provide additional diagnostic confirmation when available. In the present case, the diagnosis was supported by characteristic morphological features and MDM2 and CDK4 immunoreactivity; however, amplification of these genes was not confirmed by FISH, representing a limitation of the case.

Complete surgical resection with negative margins remains the cornerstone of treatment for localized disease. Nevertheless, negative surgical margins do not preclude early systemic progression in high-grade DDLPS, as illustrated by the rapid development of pulmonary and pleural metastatic disease in our patient shortly after surgery. The role of radiotherapy and systemic therapy remains individualized, particularly in the absence of evidence specific to spermatic cord DDLPS. Given the potential for aggressive progression, early recognition, comprehensive staging, multidisciplinary management, and close postoperative surveillance are essential. Further studies are needed to better define prognostic factors and establish the role of systemic and targeted therapies in patients with advanced DDLPS of the spermatic cord.

## Data Availability

The data presented in this study are not publicly available due to privacy and ethical restrictions but are available from the corresponding author upon reasonable request.

## Abbreviations

The following abbreviations are used in this manuscript:

LPS	Liposarcoma
DDLPS	Dedifferentiated liposarcoma
β-hCG	Beta-human chorionic gonadotropin
LDH	Lactate dehydrogenase
AFP	Alpha-fetoprotein
MDM2	Mouse double minute 2 homolog
CDK4	Cyclin-dependent kinase 4
STAT6	Signal transducer and activator of transcription 6
H&E	Hematoxylin and eosin
